# Biophysical assays to test cellular mechanosensing: moving towards high throughput

**DOI:** 10.1007/s12551-024-01263-w

**Published:** 2024-12-20

**Authors:** Marta Cubero-Sarabia, Anna Maria Kapetanaki, Massimo Vassalli

**Affiliations:** James Watt School of Engineering, University of Glasgow, Glasgow, UK

**Keywords:** Mechanobiology, Mechanosensitivity, Piezo1, Cell mechanics, Atomic force microscopy

## Abstract

Mechanosensitivity is the ability of cells to sense and respond to mechanical stimuli. In order to do this, cells are endowed with different components that allow them to react to a broad range of stimuli, such as compression or shear forces, pressure, and vibrations. This sensing process, mechanosensing, is involved in fundamental physiological mechanisms, such as stem cell differentiation and migration, but it is also central to the development of pathogenic states. Here, we review the approaches that have been proposed to quantify mechanosensation in living cells, with a specific focus on methodologies that enable higher experimental throughput. This aspect is crucial to fully understand the nuances of mechanosensation and how it impacts the physiology and pathology of living systems. We will discuss traditional methods for studying mechanosensing at the level of single cells, with particular attention to the activation of the mechanosensitive ion channel piezo1. Moreover, we will present recent attempts to push the analysis towards higher throughput.

## Introduction

Every day, we, as complex animals, experience the world around us using our senses, and this is crucial to our ability to organize in a social context, find food, and—ultimately—survive and grow. This ability is based on complex organs, such as the eyes, ears, and olfactory system, but all this has evolved from basic molecular components providing sensory functionality to single cells: chemoreceptors, photoreceptors, and mechanoreceptors (Schlosser 2018). The nature and mechanism of sensing for the first two classes have been widely studied during the past century, being the subjects of biochemistry and photobiology, respectively. In contrast, little is still known about the mechanosensation process at the level of single cells. The unique technical challenges posed by this mechanism have specifically slowed down its investigation. Forces must be controlled and measured with pN sensitivity within an integrated rig where the effect of the stimulus can be measured using nanoengineering tools, including advanced optical microscopy or electrophysiology (Mohammed et al. 2019). The last 20 years of mechanobiology research have enormously advanced our knowledge of the molecular mechanisms of mechanosensing in living cells, but many details are still elusive (Kung 2005), and the implications for pathology and physiology are now beginning to be addressed (Tschumperlin et al. 2018).

Moving from the biophysical characterization of single cells towards clinical analysis is a complex path that requires the technology to scale up and provide the throughput needed to overcome the biological diversity found at the population level. The field of mechanobiology is currently experiencing this transformation, and the scientific community is proposing new tools and assays to address the clinical translation of basic science concepts (Clyne et al. 2018). In this review, we highlight the importance of mechanosensitivity as a mechanobiological marker of cellular physiology and a key player in several pathological phenomena, with a specific focus on Piezo1-mediated mechanosensitivity (Ridone et al. 2019). Moreover, we discuss existing technology to study mechanosensitivity at the single-cell level, and we present recent developments towards the identification of effective high-throughput assays.

### Cellular mechanosensing

Mechanosensitivity or mechanosensation is the ability of cells to sense mechanical stimuli from their surrounding environment, and it is followed by mechanotransduction, their translation into biochemical signals (Kamkin and Kiseleva 2011; Basoli et al. 2018). These processes play a crucial role in defining cell fate, orchestrating cellular adaptation to the physical surroundings, and ultimately controlling processes such as proliferation, apoptosis, differentiation, migration, and organogenesis (Gudipaty et al. 2017; Zhou et al. 2020; Lee et al. 2021; Mammoto and Ingber 2010). Mechanosensation is essential for several physiological processes, including hearing (Russell et al. 1986) and touch (Handler and Ginty 2021), among others.

Mechanosensitive (MS) responses can be driven by a plethora of different mechanical stimuli. External active stimuli, including shear stress, pressure changes, and direct application of forces, can lead to different cell responses, as reviewed by Hamill and Martinac (2001). Moreover, the cellular microenvironment can also be sensed through active intracellular processes powered by the active actomyosin machinery, conferring to cells the ability to respond to their surroundings (Bennett et al. 2018). Therefore, cells need to express molecular components in charge of sensing and translating these stimuli (He et al. 2019), such as integrins and MS ion channels and focal adhesions (Ogneva 2013).

Responses to changes in the mechanical properties of the extracellular matrix (ECM), such as viscosity and stiffness, Cantini et al. (2019) are mostly mediated by integrins, heterodimeric cell adhesion molecules that physically link the ECM and the cytoskeleton in a selective manner to ECM components (Humphries et al. 2006). Recruiting and acting cooperatively with proteins including talin and fibronectin, integrins are part of the *molecular clutch* (Bennett et al. 2018), a complex of proteins responsible for the dynamic equilibrium between the forces generated intracellularly by actin remodeling and myosin motors and the passive presence of the ECM. The engagement of the complex increases in stiff or viscous environments, which present higher resistances (Bennett et al. 2018; Case and Waterman 2015), leading to the recruitment of talin and vinculin in a cascade that will ultimately consolidate a cellular response (Case and Waterman 2015).

Cells respond to external forces on timescales down to milliseconds (Wyatt et al. 2016), primarily associated with the activation of mechanosensitive (MS) channels. MS ion channels in bacteria and archaea (Martinac et al. 1987) are usually non-specific regarding their ion permeability, contrary to eukaryotic channels, which are selective. Recently, the first class of inherently mechanosensitive ion channels has been identified in mammalians, the Piezo channels family (Coste et al. 2010), triggering a completely new understanding of cellular mechanosensing. Piezo1 and Piezo2 are highly conserved across species. With a length of around 2800 residues, these proteins share around 40% of homology, with differences in structural features but a similar gating mechanism (Savadipour et al. 2023). Piezo channels are functional homotrimers, with a characteristic three-bladed propeller-like shape, linked to a central cap sitting on top of the pore (Ge et al. 2015). Piezo channels show peculiar dynamical features associated with an ensemble of states (Mulhall et al. 2023) and respond to external stimuli over a broad frequency range, thanks to a fast inactivation mechanism. While the channel mostly acts as a frequency filter (Lewis et al. 2017), it can transduce higher frequencies where the synchronization with the driving vibration is lost but leading to a tonal increase in the basal calcium concentration (Orapiriyakul et al. 2020). With a high affinity for calcium ions, Piezo1 gating initiates a plethora of physiological responses, Coste et al. (2010), including cell migration and bone formation (Zhou et al. 2020; Mousawi et al. 2020) as well as being involved in pathogenic states, such as pancreatitis (Swain et al. 2020) or cancer processes (Mousawi et al. 2020; Swain et al. 2020).

Added to the effects driven by the extracellular environment, the mechanics and tensional state of the cell also affect intracellular responses (Grandy et al. 2023) in a feedback mechanism that allows for strict and complex regulation of mechanosensation (DeBelly et al. 2022). This regulation is also achieved by the complex interaction of different cellular components; for instance, the cytoskeleton is essential for force transmission to the channel and regulates its activation threshold (Cox et al. 2016), while membrane compositions, specifically cholesterol and fatty acids, affect membrane tension and the response to mechanical stimuli (Ridone et al. 2020; Romero et al. 2019).

## Measuring cellular mechanosensation

The centrality of Piezo1-driven cellular mechanosensation is currently not matched by a suitable assay to measure it. Several biophysical methods have been proposed to directly probe the activation of Piezo1 upon mechanical stimulation. Nevertheless, a proper high-throughput cellular mechanosensitivity assay is still missing. This section briefly recapitulates the main methods to measure Piezo1 activity, presenting some promising attempts in the direction of an increased measurement yield, where the response of single cells—more than of single channels—is being monitored. The current technological scenario is discussed in view of a possible extension of current approaches towards a higher throughput assay for single-cell mechanosensitivity.

**Fig. 1:**
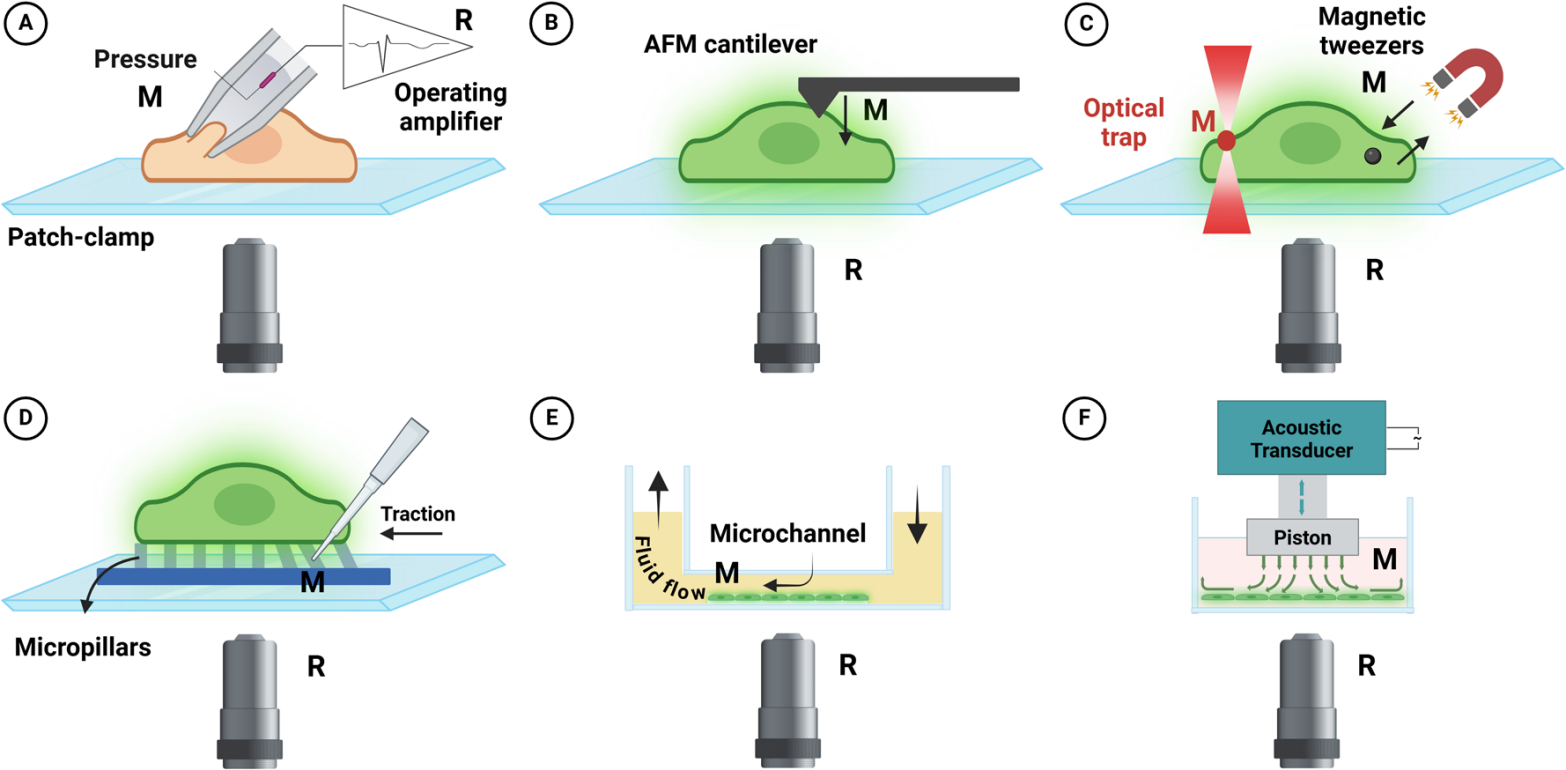
Illustration of the different techniques used for cell mechanosensitivity assessment, indicating the mechanbical stimulus M and the readout of activation R. **A** Patch-clamp in pressure control configuration. M, pressure within the pipette; R, ionic current. **B** Atomic Force Microscopy (AFM). M, force applied by the cantilever; R, fluorescence of a reporter sensing calcium concentration. **C** Remotely actuated beads, such as in optical (left) or magnetic (right) tweezers. M, force applied to the bead; R, fluorescence. **D** Micropillars. M, displacement of an elastic pillar with known compliance; R, fluorescence. **E** Microfluidic-based techniques. M, shear flow; R, fluorescence. **F** Hydrostatic pressure. M, pressure provided by a piston; R, fluorescence (image created with BioRender.com)

### Biophysical methods to measure the activation of mechanosensitive ion channels

Measuring mechanosensation at the level of single cells, as associated with the activation of MS ion channels, requires the ability to (i) aim single cells (typically over an inverted microscope), (ii) apply a controlled mechanical stimulus M, and (iii) at the same time measure the activation of MS ion channels through a readout mechanism R (see Fig. 1). The sensitivity requirements and technical complexity of this combination have slowed down the study of MS ion channels till recently.

The gold standard approach to studying mechanosensitivity has been patch clamp electrophysiology (Fig. 1A). This technique, first described by Neher and Sakman (Neher and Sakmann 1976), has been widely used since the first reports of mechanosensitive channels (Martinac et al. 1987). Electrophysiology, based on measuring the ionic current across the cell membrane, can be adapted to study mechanosensitive ion channels in different configurations. The mechanical stimulus can be external, provided by a motorized needle, while the current is monitored with a pipette, either in a whole-cell configuration (measuring the contribution of all channels in the plasma membrane) or in patch mode (where the current is associated with the few channels in the $\mu$m-size patch). Alternatively, the mechanical stimulus can be applied through the patch pipette itself by imposing pressure and stretching the membrane while measuring the current (Fig. 1A). This approach offers several advantages in terms of sensitivity and specificity, and its application to the study of MS ion channels has been extensively reviewed (Kornreich 2007; Zhao et al. 2008; Lhomme et al. 2019; Gaub and Müller 2017).

More recently, the integration of atomic force microscopy (AFM) and fluorescence microscopy has been suggested as a powerful method to study MS ion channels. An AFM-based set-up was designed in order to elucidate the activation mechanism of Piezo1 by combining AFM with confocal microscopy and using calcium imaging to report Piezo1 activation (Fig. 1B). This seminal paper highlighted the importance of the ECM-mediated adhesion to activate the channel during pulling (Gaub and Müller 2017). Due to the relatively widespread availability of bio-AFM systems integrated with an optical microscope, this method has been successfully replicated to study different systems, such as evaluating the connection of mechanosensitivity and cancer (Yamagishi et al. 2021; Weng et al. 2018). Interestingly, the role of substrate mechanics has been explored, showing environment-mediated modulation of mechanosensitivity in cardiac fibroblasts (Braidotti et al. 2024). The interesting aspect of AFM is that it allows a direct measurement of the force while monitoring the response of cells maintained at 37 degrees, which is difficult using electrophysiology-based methods.

Fluidic Force microscopy (Li et al. 2021) has been recently proposed as a promising method to study mechanosensitivity (Lüchtefeld et al. 2024). This technique is based on the use of force-controlled hollow cantilevers, in fact combining the advantages of both AFM (force control, working in physiological conditions) and micropipettes (fine control of the pressure and the ionic composition of the solution).

Other than AFM, several other single techniques have been proposed, where the mechanical stimulus can be introduced using a device capable of applying forces to single cells, such as optical tweezers (Fig. 1C left), magnetic Tweezers (Fig. 1C right), micropillars (Fig. 1D), or glass needles (Capponi et al. 2022) where the activation of the channel is measured by means of a fluorescence calcium reporter that gets activated by the opening of the channels and the consequent increase in cellular $\mathrm {Ca^{2+}}$concentration. These efforts have been reviewed elsewhere (Sun et al. 2021; Roeterink et al. 2024). While these biophysical methods offer the opportunity to study the response of single cells to controlled mechanical stimulation with high resolution and sensitivity, they lack the throughput and usability required to translate this concept to clinically and physiologically relevant applications.

### Flow-based assays

Piezo1 responds to shear flow, inducing $\mathrm {Ca^{2+}}$entry in the cell. This observation suggests a sensible strategy to assess Piezo1 mechanosensitivity by integrating controlled fluid shear stress and calcium imaging. This basic idea has been implemented in different configurations, incorporating flow shear stress, compression stimuli, or both (see Fig. 1 E, F).

The simplest configuration for testing the response of Piezo1 to shear can be obtained by culturing cells within a transparent microfluidic chamber mounted on a fluorescent microscope. Loading the cells with a calcium reporter, such as Fluo4-derived probes, and inducing a step change in the flow rate, the mechanosensitivity of the cellular system can be measured in terms of fluorescence increase (Fig. 1E). This approach was implemented by Maneshi et al. using a piezoelectric actuator to apply fast flow steps on the cells (Maneshi et al. 2015) and used as an assay to identify blockers of Piezo1, showing that A$\beta$ monomers inhibit Piezo1-mediated shear flow sensitivity (Maneshi et al. 2018). The advantage of this configuration is that the cells can be seeded on a coated substrate (as far as the thickness does not impair the optical access), and this feature has been used to demonstrate that the mechanosensitive response of Piezo1 depends on the elements of the extracellular matrix (Lai et al. 2022). A key aspect is the calibration of the flow imparted to the cells, and the technical details of the various implementations have been reviewed elsewhere (Maneshi et al. 2017). Interestingly, a low-cost version of this set-up has been proposed, based on a 3D-printed dynamic gravity pump which does not require expensive flow control devices to be integrated into the device (Concilia et al. 2022).

Other than shear flow, living cells in a physiological environment can be exposed to pressure changes or more complex turbulent regimes. To achieve this condition, a different configuration can be designed using pistons or pressurized membranes. An interesting 384-well high-throughput shear stress stimulation system has been proposed by Xu et al. (2018) to study mechanosensitive G-coupled protein receptors in the vasculature. In this case, the shear flow was applied by an oscillatory moving piston working on a 348-well system, effectively improving the throughput to 348 parallel conditions (Fig. 1F). Interestingly, the system was tested in human umbilical vein endothelial cells (HUVECs) in siRNA-Piezo1 transfected cells, observing the lack of induced calcium response compared to wild-type (WT) cells. A variation of this system was later proposed by Strittmatter et al. (2021), who studied different stimulation regimes, possibly leading to turbulent flow. Interestingly, the authors extended the use of the device over longer terms, evaluating both the short-term response (calcium influx) and the longer-term change in gene expression.

Shear flow stimulation chambers with integrated optical access offer an exciting platform for the screening of cellular mechanosensitivity. The possibility of coating the substrate allows the manipulation of the tensional state of the cells and the integration of different components of the extracellular matrix. However, the set-up results in a relatively bulky system, which is tricky to directly integrate within a standard optical microscope (if additional imaging is required). Moreover, the flow experienced by each single cell will depend on the orientation and position of the cell within the culture, which increases the variability and limits the quantification of the measurements. This cannot be easily overcome by the statistics as the number of cells imaged by the device is limited by the field of view and compromises with image quality (which would reduce the ability to isolate the response of single cells in the population). To achieve a more detailed understanding of cellular mechanosensitivity, a proper single-cell high-throughput analysis is envisaged, as discussed in the next paragraph.

### Towards high-throughput mechanosensitivity assays

The study of ion channels is central to pharmacology, and the development of automated patch clamp (APC) devices (Obergrussberger et al. 2020) has sparked from academia to involve big industries and contract research organizations in search for a high-throughput drug screening solution (Rogers et al. 2024).

Extending this approach to MS ion channels, Barthmes et al. have adapted a commercial small automated planar patch clamp system (Brüggemann et al. 2008) to study bacterial MS ion channels. In the proposed configuration, a pressure difference between the inside and the outside of the cell could be controlled with a pump, inducing a tuneable volume change while measuring the corresponding current activation. More recently, Murciano et al. have extended the idea to study Piezo1 with a commercial APC system (Murciano et al. 2023). In this paper, a mechanical stimulation protocol was proposed whereby cells trapped on the pore (subjected to electrophysiological recording) were approached with the pipette and exposed to a blow of solution dispensed at a high flow rate, in sync with a triggered electrical recording. Single cells were exposed to two intertwined mechanical stimuli, direct pressure, and shear stress, that the authors calibrated.

While the throughput is still limited to a few 100 cells per experiment, the usability and automation of these platforms largely outperform the standard laboratory patch clamp electrophysiology set-up, opening towards drug screening applications. However, the specific geometry of the APC devices precludes any manipulation of the local microenvironment (such as having substrates with controlled mechanical properties), and this could limit the investigation of mechanosensitivity phenomena.

When it comes to pushing the boundaries of experimental throughput while keeping the information at the single-cell level, microfluidics is the key technology (Yin and Marshall 2012). In the area of mechanobiology, microfluidics has been effectively adopted to scale up the characterization of the cellular mechanotype (Urbanska and Guck 2024). A few devices measuring cellular deformability have been proposed, either using constrictions, concurrent flows, or hydrodynamic deformation (these are reviewed in Urbanska et al. (2020)). This breakthrough technology has been expanded to integrate the measurement of visco-elastic properties (Reichel et al. 2024) and linked to a sorting unit to select cells based on their mechanical phenotype (Faigle et al. 2015; Chen et al. 2024). The increase in throughput offered by microfluidics allowed the scientific community to address several clinically relevant questions, raising huge expectations for the translational potential of the mechanical phenotype (Kozminsky and Sohn 2020), showing significant differences even for adherent cell types after careful separation from the tissue of origin (Soteriou et al. 2023).

Deformation cytometry is heavily based on fast image recognition, and it can be smoothly integrated with fluorescence imaging (Nawaz et al. 2020), with the potential to be extended towards measuring mechanosensitivity. However, the time scale of the biochemical response to mechanical stimuli spans a broad range, from the ms regime of the immediate channel gating, to the few s required for the cells to fully interpret the message and respond, and this added complexity has so far hindered the development of a proper microfluidic-based mechanosensitivity assay.

## Conclusions

The study and understanding of mechanosensation and the underlying mechanisms of mechanosensitivity still have a long path ahead. In this short review, we have highlighted the importance of directly measuring mechanosensitivity to understand the complex problem of mechanoadaptation, which is involved in numerous fundamental physiological and pathological processes.

A particular area of interest is related to Piezo1/2 physiology. These channels are ubiquitous in the organism, across species and kingdoms (Ridone et al. 2019), and their ability to respond to mechanical stressors potently depends on the mechanical properties of the microenvironment (membrane, cytoskeleton, extracellular matrix), alluding to the existence of a general mechanism of mechanical feedback whereby the sustained activation of Piezo1 induces cytoskeleton rearrangement (Geng et al. 2021; Morena et al. 2024) and matrix remodelling (Kelley et al. 2023), tuning the local mechanical phenotype and thus the mechanosensitivity, and bringing the biological system outside of the homeostatic equilibrium when it becomes aberrant (Chen et al. 2018; He et al. 2024).

We envisage scaling up the existing technology as a significant step towards the understanding of mechanoadaptation, combining high-throughput measurements and fine control of the local microenvironment, and we expect microfluidics to play a central role in this process.

## Data Availability

No datasets were generated or analyzed during the current study.
